# Migration distance does not predict blood parasitism in a migratory songbird

**DOI:** 10.1002/ece3.5404

**Published:** 2019-07-01

**Authors:** Marjorie C. Sorensen, Tanmay Dixit, Kevin J. Kardynal, Jason Newton, Keith A. Hobson, Staffan Bensch, Susanne Jenni‐Eiermann, Claire N. Spottiswoode

**Affiliations:** ^1^ Department of Zoology University of Cambridge Cambridge UK; ^2^ Department of Integrative Biology University of Guelph Guelph Ontario Canada; ^3^ Environment and Climate Change Canada Saskatoon Saskatchewan Canada; ^4^ NERC Life Sciences Mass Spectrometry Facility Scottish Universities Environmental Research Centre East Kilbride UK; ^5^ Department of Biology University of Western Ontario London Ontario Canada; ^6^ Department of Biology Lund University Lund Sweden; ^7^ Swiss Ornithological Institute Sempach Switzerland; ^8^ DST‐NRF Centre of Excellence at the FitzPatrick Institute of African Ornithology University of Cape Town Cape Town South Africa

**Keywords:** disease ecology, malaria parasites, migration, Palearctic–African songbird

## Abstract

Migration can influence host–parasite dynamics in animals by increasing exposure to parasites, by reducing the energy available for immune defense, or by culling of infected individuals. These mechanisms have been demonstrated in several comparative analyses; however, few studies have investigated whether conspecific variation in migration distance may also be related to infection risk. Here, we ask whether autumn migration distance, inferred from stable hydrogen isotope analysis of summer‐grown feathers (*δ* ^2^H_f_) in Europe, correlates with blood parasite prevalence and intensity of infection for willow warblers (*Phylloscopus trochilus*) wintering in Zambia. We also investigated whether infection was correlated with individual condition (assessed via corticosterone, scaled mass index, and feather quality). We found that 43% of birds were infected with *Haemoproteus palloris* (lineage WW1). Using generalized linear models, we found no relationship between migration distance and either *Haemoproteus* infection prevalence or intensity. There was spatial variation in breeding ground origins of infected versus noninfected birds, with infected birds originating from more northern sites than noninfected birds, but this difference translated into only slightly longer estimated migration distances (~214 km) for infected birds. We found no relationship between body condition indices and *Haemoproteus* infection prevalence or intensity. Our results do not support any of the proposed mechanisms for migration effects on host–parasite dynamics and cautiously suggest that other factors may be more important for determining individual susceptibility to disease in migratory bird species.

## 1 INTRODUCTION

The seasonal movements of migratory animals have important ecological consequences (Bauer & Hoye, 2014) including animal host–parasite interactions and the spread of disease (Altizer, Bartel, & Han, 2011). Understanding how individual variation in migration distance may be associated with the risk of infection is important given that migration distances for migratory bird species will change in response to a changing climate (Barbet‐Massin, Walther, Thuiller, Rahbek, & Jiguet, 2009; Doswald et al., 2009; Visser et al., 2009). While there have been several comparative analyses of migration effects on levels of parasitism, few studies have investigated whether conspecific variation in migration distance may also be related to infection risk (but see Kelly et al., 2016).

Migration may increase infection risk via reduced investment in immune defense during long migratory flights, or via increased exposure to parasites. Migratory flight is an energetically demanding period of the annual cycle, with important trade‐offs between flight and immune function investment (Eikenaar & Hegemann, 2016; Nebel et al., 2012; Owen & Moore, 2008; but see Hasselquist, Lindstrom, Jenni‐Eiermann, Koolhaas, & Piersma, 2007). Individuals traveling longer distances between breeding and wintering grounds may be forced to allocate more energy to flight and away from immune defense, resulting in compromised immunity, heightened susceptibility to infection, and accordingly increased levels of parasitism. Migratory birds also move across large distances and through different habitat types during migration, which may increase encounters with more abundant or diverse parasite assemblages and so result in increased levels of parasitism (Altizer et al., 2011; Gutiérrez, Rakhimberdiev, Piersma, & Thieltges, 2017; Teitelbaum et al., 2018). In contrast, migration may also influence host–parasite dynamics by removing infected individuals from the population during strenuous journeys (migratory culling; Bradley, 2005) or by allowing migrants to escape harmful parasites (Hall & Bartel, 2014). These interactions are complex, and comparative studies from various taxa have shown that migratory species can both have higher (Koprivnikar & Leung, 2014; Leung & Koprivnikar, 2016; Teitelbaum et al., 2018) and lower (Satterfield et al., 2015) levels of parasitism than resident species.

Malaria (*Plasmodium*) and related haemosporidian parasites (*Haemoproteus* and *Leucocytozoon*) are common, species‐rich and globally distributed across bird species (Lapointe et al., 2012). The disease is characterized by an acute short‐lived stage, after which most survivors maintain chronic infections that vary in low‐level intensity (Asghar et al., 2012). The short‐term fitness costs of chronic haemosporidian infections are not always clear (Asghar et al., 2015; Lapointe et al., 2012); however, recent evidence suggests that the consequences of low‐intensity infection may only become observable over the long term via reduced lifespan and lifetime reproductive output (Asghar et al., 2015). Understanding the factors influencing individual infection risk is important given the long‐term costs of chronic avian malaria infections.

Here, we investigated whether migration distance, inferred from stable hydrogen isotope values in summer‐grown feathers (*δ* ^2^H_f_), was correlated with malaria prevalence and intensity of infection for willow warblers (*Phylloscopus trochilus*) wintering in Zambia. Hydrogen in feathers is ultimately derived from environmental waters, especially those associated with precipitation (Hobson & Wassenaar, 2008). Stable isotopes of hydrogen are passed through the food web, and feathers have been found to be a good indicator of mean amount‐weighted growing season or annual precipitation *δ* ^2^H values (*δ* ^2^H_p_) that are fixed once formed. In western Europe, *δ* ^2^H_f_ isoclines are largely latitudinal (i.e., east–west), and so *δ* ^2^H_f_ values act as a proxy for migration distance to wintering grounds. Willow warblers are one of the most abundant Palearctic–African migratory songbird species. They are a small (8 g) passerine species that breeds widely in temperate Europe and Asia and winters throughout sub‐Saharan Africa (Cramp & Perrins, 1994). In Zambia, willow warblers arrive from mid‐ to late September after autumn migration, without long‐term staging sites en route (Dowsett, 2009). Migration distance may vary substantially across the study population, given that individuals may breed at different distances from the Zambian winter site. Two lineages of malaria‐like parasites (WW1 and WW2), belonging to the morphospecies *Haemoproteus palloris* (Dimitrov et al., 2016) and *H. majoris* (Križanauskienė et al., 2006), respectively, have previously been recorded as the dominant haemosporidian parasites in willow warblers (WW1 and WW2; Bensch & Akesson, 2003).

This study had two objectives. First, we investigated the relationship between migration distance and malaria infection. If infected individuals are unable to migrate longer distances (migratory culling; Bradley, 2005; Hall & Bartel, 2014), then a negative relationship is expected between migration distance and the prevalence and intensity of malaria infections on African wintering grounds. Alternatively, if trade‐offs between migration and immunity compromise immune investment during migration, or if longer migrations increase parasite exposure (Nebel et al., 2013; Teitelbaum et al., 2018), then a positive relationship is expected between migration distance and the prevalence and intensity of infections. Second, we investigated the relationship between malaria infection and individual condition. If the costs of low‐intensity chronic malarial infection only become observable over an individual's lifetime (Asghar et al., 2015), then no relationship is expected between short‐term measures of individual condition (estimated from circulating corticosterone, scaled mass index, and feather quality) and malaria prevalence or intensity of infection (Schoenle et al., 2017; Sorensen et al., 2016). Understanding the factors that predict individual infection risk is an important component of effectively predicting the population dynamics of species, especially given that global change is rapidly affecting some of the mechanisms hypothesized to affect infection risk in migratory animals (Garamszegi, 2011; López‐Calderón et al., 2018).

## 2 METHODS

### 2.1 Field site and study species

Fieldwork was carried out on Muckleneuk Farm, near Choma, southern Zambia (16°39′S, 27°00′E), in ca. 900 ha of thornbush and miombo woodland, during October–December 2012. Willow warblers were captured daily from 06:00 to 08:30 hr, by using willow warbler song (alternating between three songs recorded in Zambia) to entice birds toward mist nets.

Immediately after capture, a ~50 μl blood sample was taken from each individual. Handling time (i.e., the mean handling time between birds hitting the net and blood collection) was 3.1 min (range 2.5–3.8 min). Blood was collected with heparinized capillary tubes. One drop of blood was transferred to a slide, and a blood smear of one cell layer thick was made. The remaining blood was transferred to a 1.5‐ml Eppendorf tube and centrifuged within 4 hr. Plasma and red blood cells were stored at −20°C until further analysis. The fifth rectrix feather was plucked and stored dry in ziplock plastic bags for stable isotope analysis. Wing, total length of bill and head, and tarsus were measured using digital calipers (±0.1 mm). Body mass was measured using an electronic balance (±0.1 g). As an estimate of body condition, we calculated scaled mass index (Peig & Green, 2009), which scales the mass of all individuals to that expected if they were all of identical body size. We used tarsus length as a single measure of structural size since it was correlated most strongly with body mass (*r* = 0.29, *p* = 0.02), and a principal components analysis (PCA) of multiple size measures complicates the interpretation of scaling relationships between body mass and linear size measures (Peig & Green, 2009). Sex was determined using molecular methods following (Griffiths, Double, Orr, & Dawson, 1998).

### 2.2 Malaria prevalence and intensity

A polymerase chain reaction (PCR) was used to determine the prevalence and strain of parasites in the genera *Plasmodium* and *Haemoproteus* infecting willow warblers wintering in Zambia. We expected to find either WW1 (*H. palloris*) or WW2 (*H. majoris*). WW1 is very likely transmitted in Africa, since juveniles sampled in Europe do not carry WW1 prior to autumn migration, whereas WW2 is transmitted on European breeding grounds (Bensch & Akesson, 2003). Willow warbler DNA was extracted using Qiagen^TM^ extraction kits. We screened birds for parasites by amplifying with PCR a 525‐bp fragment of the parasite cytochrome *b* gene using the primers HAEMF and HAEMR2 (Bensch et al., 2000). This protocol has been demonstrated to be more efficient than alternative PCR‐based protocols and traditional smear analyses (Richard et al., 2002). Direct sequencing was done with the HAEMF primer of all positive reactions and loaded on an ABI PRISM^TM^ 310 sequencing robot. To determine the strain of infection, electropherograms were manually inspected and the proofread sequences were matched with sequences held in MalAvi (Bensch, Hellgren, & Perez‐Tris, 2009).

To determine malaria intensity, we analyzed blood smears from all individuals with positive PCRs under microscopic examination. Slides were examined with 1,000× magnification (oil immersion) counting the number of parasites (previously identified via PCR) in 7,500–8,000 red blood cells in an area of the blood smear with homogenous dispersion of cells (Hasselquist, Ostman, Waldenstrom, & Bensch, 2007). The number of red blood cells was estimated as the mean of the first and last field, multiplied by the number of fields examined (Hasselquist, Ostman, et al., 2007). Previous work has shown this measure to be highly correlated with infection intensity measured through quantitative PCR (Zehtindjiev et al., 2008). Infected red blood cells were found in all blood smears from PCR‐positive reactions. Parasite intensity is reported as the proportion of host red blood cells infected.

### 2.3 Corticosterone analysis

High levels of circulating corticosterone can indicate compromised individual condition (Wingfield & Ramenofsky, 1999). Plasma corticosterone concentration was determined using an enzyme immuno‐assay following (Jenni‐Eiermann et al., 2015). In short, corticosterone in 5 µl plasma and 195 µl water was extracted with 4 ml dichloromethane, redissolved in phosphate buffer and given in duplicates in the enzyme immuno‐assay. The dilution of the corticosterone antibody (Chemicon; cross‐reactivity: 11‐dehydrocorticosterone 0.35%, progesterone 0.004%, 18‐hydroxydeoxycorticosterone 0.01%, cortisol 0.12%, 18‐hydroxycorticosterone 0.02%, and aldosterone 0.06%) was 1:8,000. The concentration of corticosterone in plasma samples was calculated by using a standard curve run in duplicate on each plate. Plasma pools from chickens with two different corticosterone concentrations were included as internal controls on each plate. Intra‐assay variation for the low and high chicken plasma controls was 7.9% and 11.9% and interassay variation 17.7% and 5.16%, respectively. Corticosterone concentrations did not vary according to handling time (*r* ^2^ = 0.002, *p* = 0.77).

### 2.4 Feather quality

Feather quality can be indicative of individual condition (Carbonell & Tellería, 2010; DesRochers et al., 2009). We quantified rectrix quality through four parameters: feather mass, rachis width, barbule number, and interbarb distance. The dry mass of each feather was measured using a digital balance (±0.1 mg). Rachis width was measured at the base of the vane with digital calipers (±0.01 mm). Barbule numbers and interbarb distances were measured on images taken using an AxioCam MRC Zeiss camera mounted on a Leica MZ 95 dissecting scope. Barbules were counted along a 1.5 mm length for three barbs in each feather. Standardized 1.5 mm lengths were traced in ImageJ. The mean across barbs was used for analyses. Ten interbarb distances were measured for each feather, beginning at the rachis. Because we were interested in a general feather quality measure, we used PCA to derive composite feather quality scores for each individual. Variables were log‐transformed and centered prior to PCA. All four variables loaded strongly on the PC1 axis (PC1 = 42% of variation), with rachis width having the largest effect (rachis width: 0.6, feather weight: 0.52, barbule number: 0.46, interbarb distance: 0.39). PC1 was used in all subsequent analyses.

Willow warbler flight feathers are replaced prior to autumn migration (Cramp & Perrins, 1994), and given that it is unknown whether infected individuals acquired infection during the autumn migration preceding the study or during a previous year, it is likely that not all birds carrying infection in Zambia were infected during feather replacement. Therefore, feather quality is likely a weaker indicator of individual condition at the exact time of malarial infection.

### 2.5 Hydrogen isotopes

Several recent studies have utilized the latitudinal gradient of *δ* ^2^H in amount‐weighted growing season precipitation across the Western Palearctic to estimate the latitude at which inert tissues are grown (Arizaga et al., 2016; Bowen, Wassenaar, & Hobson, 2005; Caizergues, Wilgenburg, & Hobson, 2016; Guillemain, Wilgenburg, Legagneux, & Hobson, 2013; Procházka et al., 2013). However, since the majority of studies that use *δ* ^2^H_f_ as an estimate of latitude have relied on the stronger North America latitudinal gradient in *δ* ^2^H_f_, we used the relationship between body size and *δ* ^2^H_f_ to confirm the applicability of this method for willow warblers. In general, willow warbler body size increases with breeding latitude (Bensch, Andersson, & Akesson, 1999). As an index of body size, we used the first principal component scores (PC1) from wing length, tail length, and body mass since these size measures were found to correlate with latitude in willow warblers (Bensch et al., 1999). All three variables loaded strongly on the PC1 axis (PC1 = 44% of variation), with wing length having the largest effect (body mass: 0.52, wing: 0.69, tail: 0.50). We did not include size as an additional geomarker in our estimate of migration distance (Rushing et al., 2014) since georeferenced body size information was not available for willow warblers.

Prior to isotope analyses, feathers were washed in 2:1 chloroform: methanol solution, then rinsed with distilled water, and left to air dry for 24 hr. Cleaned samples of approximately 0.2 mg were weighed into silver capsules and pyrolyzed in an Elementar PyroCube elemental analyzer over glassy carbon (1,350°C). The resulting H_2_ was admitted into the source inlet of a Thermo XP Plus mass spectrometer. Measurements are reported in *δ*‐notation relative to the international standard VSMOW (Vienna Standard Mean Ocean Water). Organic materials involving H not bonded to carbon will readily exchange a portion of H with ambient (i.e., laboratory) water vapor (Schimmelmann et al., 1993). Subtraction of the effect if this exchangeable hydrogen is attained using standards of similar matrix with known nonexchangeable (i.e., indigenous) hydrogen isotope compositions via comparative equilibration (Wassenaar & Hobson, 2003). In our case, these were CFS (chicken feathers—148.61‰), BWB‐II (bowhead whale baleen—109.51‰), and ISB (black‐legged kittiwake feathers—68.8‰; for CFS and BWB‐II, see Hobson & Wassenaar, 2008; for ISB see Fox, Christensen, Bearhop, & Newton, 2007). Within‐run replicate measurements (*n* = 6) of these standards implied measurement errors (*SD*) of around 2‰ for δ^2^H.

### 2.6 Assignment to origin analysis

We used a spatially explicit likelihood assignment method to define probable winter origins of willow warblers (Hobson et al., 2009; Royle & Rubenstein, 2004). To this end, we converted an amount‐weighted growing season precipitation surface (δ^2^H_p_) for Eurasia (Bowen et al., 2005) to a feather isoscape using the calibration for juvenile reed warblers (*Acrocephalus scirpaceus*) from Procházka et al. (2013) (−10.29 + 1.28*δ^2^H_p_). We used this equation because we lacked a similar one for willow warblers; however, both species have similar diets and migratory behaviors (i.e., insectivores and long‐distance migrants), and thus, we expected use of this calibration to provide reasonable results. We used the standard deviation of the residuals of the linear regression model (*SD* = 10.36‰) from Procházka et al. (2013) as an estimate of error in the assignments. We downloaded digital range maps from BirdLife International and NatureServe (2011) in order to restrict origin assignments to the willow warbler breeding range. Recent research using genotyping has shown that one of three recognized willow warbler subspecies, *P. t. trochilus* (Cramp & Perrins, 1994)*,* does not winter in Zambia (S. Bensch unpublished data); therefore, we excluded this region of the breeding range (Ireland east to Slovakia and eastern Poland, central Norway and Sweden to Spain, France, and northern Hungary) from assignment to origin analyses.

We applied a 2:1 odds ratio to assign individuals to potential breeding ground origin, where raster cells (pixels) in the isoscape in the upper 67% of probabilities were considered as likely origins (1) and all others were considered unlikely origins (0). Assignments conducted for feather samples resulted in a spatially explicit binary surface (assignment raster) for each individual, which were summed across all individuals to represent potential origins for that species. Manipulation of digital files and assignment to origin analyses were conducted using multiple packages including “raster” 2.5‐8 (Hijmans, 2016) and “maptools” 0.8‐39 (Bivand & Lewin‐Koh, 2017) in the R statistical computing environment 3.5.0 (R Core Team, 2018).

We contrasted the assignment to origin maps of birds with and without malaria, to determine whether they varied spatially. To this end, we scaled each assignment raster to one and used Dutilleul's, Clifford, Richardson, and Hemon (1993) modified *t* test to assess the significance of Moran's *I* correlation coefficient between the spatial processes of each assignment raster. This analysis was conducted using the “SpatialPack” package in the R environment 3.5.0 (Osorio et al., 2016). Further, we estimated migration distances (Vincenty, 1975) from the wintering site to the centroid of the area of potential origin from the probabilistic assignments, using δ^2^H_p_ for each individual. Distance between wintering and probable breeding ground centroids was calculated using the “geosphere” package (Hijmans, 2016).

### 2.7 Statistical analysis

All analyses were restricted to males (*n* = 64), as the number of females caught and sampled (*n* = 4) was too low to include in statistical models. All analyses were conducted in R ver. 3.5.0 (R Core Team, 2018). We included date of capture (birds were sampled over 44 days in Zambia) as a covariate in all models, since birds sampled earlier in the wintering season may have different infection or individual condition profiles than birds sampled later in the season. To test for predictors of parasitemia (infected vs. noninfected individuals determined via PCR), we used a generalized linear model with a binomial error distribution (using a logit link function). To test for predictors of parasite intensity (determined via blood smears), we used a generalized linear model with a quasi‐Poisson error distribution (using a logarithmic link function) as appropriate for proportional response variables and allowing for overdispersion (O'Hara & Kotze, 2010). To maximize sample size, separate models were run for migration distance and body condition indices.

## 3 RESULTS

### 3.1 Prevalence, intensity, and strain of infection

The prevalence of *Haemoproteus* infection for overwintering, male willow warblers in Zambia was 43%. Most infections were at chronic levels of intensity, though three birds had intensities >3%, suggesting primary or recent relapse infections (mean = 0.99% infected erythrocytes, range = 0.01%–9.22%; Hasselquist, Ostman, et al., 2007). Sequencing showed that every infected bird (*n* = 26) carried the lineage WW1. No birds were infected with WW2.

### 3.2 Feather *δ* ^2^H and assigned breeding origins

**Figure 1:**
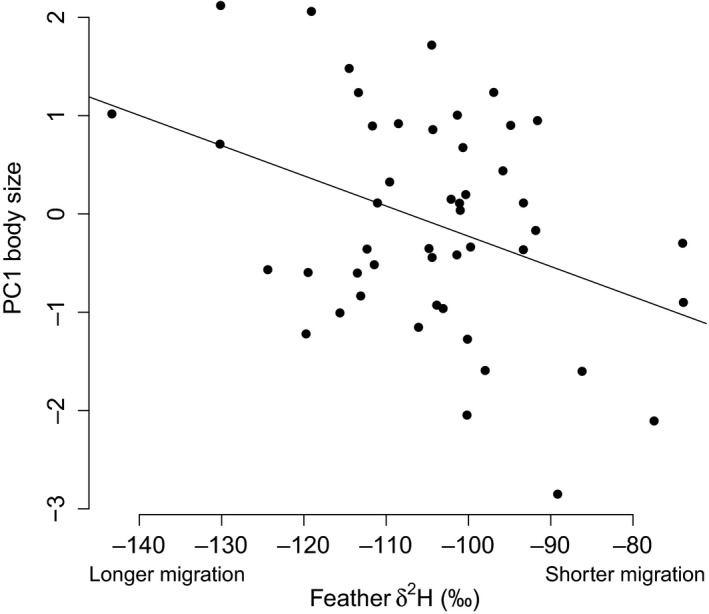
Relationship between willow warbler body size (PCA of wing length, body mass, tail length) and *δ* ^2^H_f_ (*r* ^2^ = 0.15, *p* = 0.007, *n* = 48) from feathers grown on the breeding grounds

We found a negative relationship between individual body size (PC1: weight, wing, tail) and *δ* ^2^H_f_ (*r* ^2^ = 0.15, *p* = 0.007, Figure 1), such that larger individuals originated from higher latitudes than smaller individuals, adding further support to *δ* ^2^H_f_ as a reliable indicator of breeding/natal latitude in this species.

**Figure 2:**
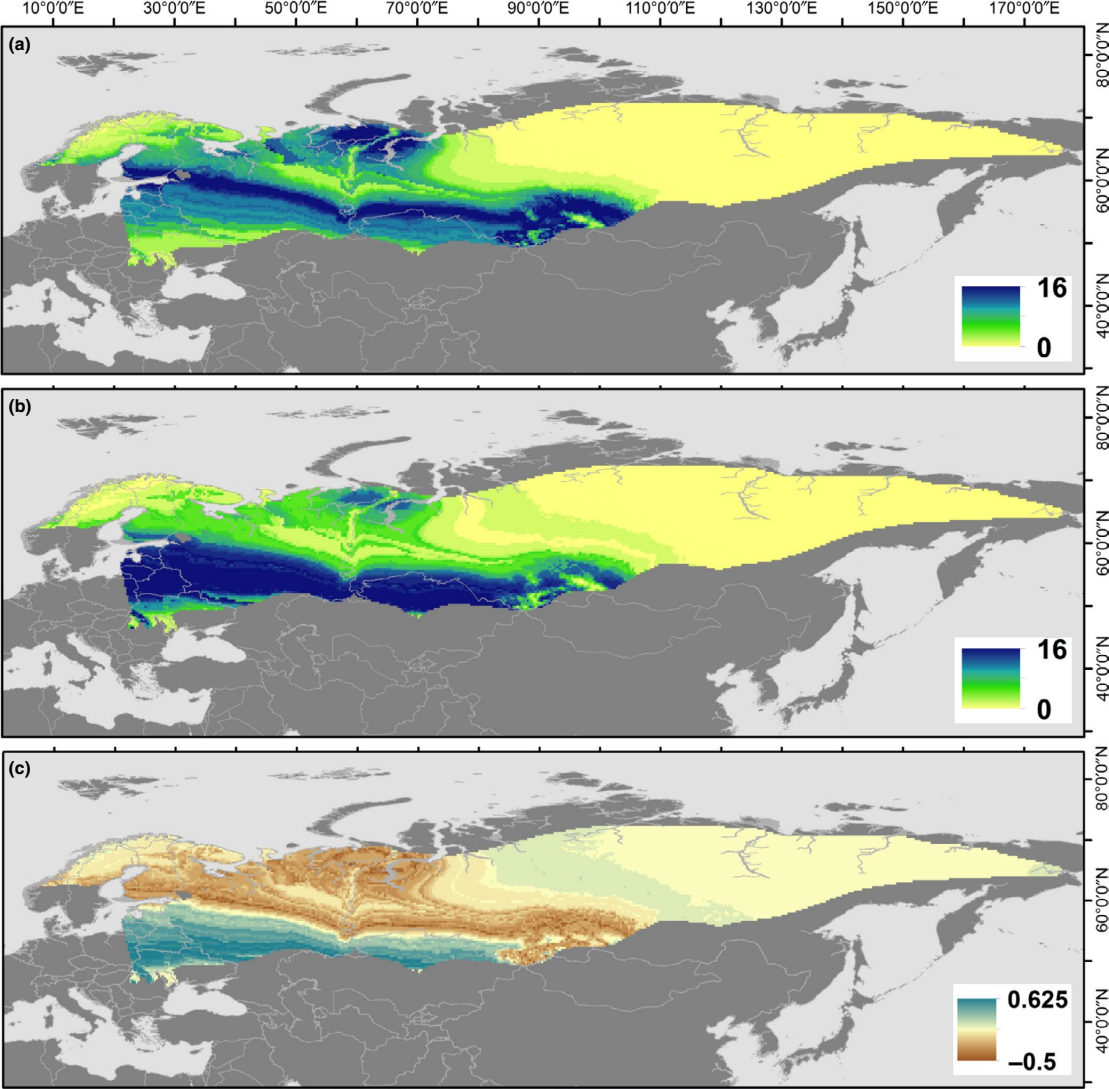
Potential origins of willow warblers from Northern Hemisphere breeding grounds, within the breeding range of *Phylloscopus trochilus acredula and P. t. yakutensis* for (a) infected and (b) noninfected birds. The range of the subspecies *P. t. trochilus* (mainly southern Scandinavia and western Europe) was excluded from assignment analyses based on genotype evidence from the Zambian study population (S. Bensch unpublished data). Assignments based on *δ* ^2^H_f_ (‰) were determined using a maximum‐likelihood approach (see Methods2). The legends (a, b) indicate the number of male birds potentially originating from a particular cell in the *δ* ^2^H_f_ isoscape. Differences in origins between scaled assignment rasters (spatially explicit binary surface) of infected and noninfected willow warblers are shown in (c), where values ≈ −0.5 (brown) indicate a higher proportion of infected birds potentially deriving from those areas, values ≈ 0.625 (blue) indicate higher proportions of noninfected birds, and values ≈ 0 (yellow) indicate little or no difference in the proportion of individuals deriving from those cells. The observed spatial difference in the origins of infected versus noninfected birds resulted in only a small difference in migration distance (~214 km; see Methods2)

The *δ* ^2^H_f_ of male willow warblers wintering in Zambia ranged from −143.3‰ to −73.9‰ (*n* = 47). Willow warblers wintering in Zambia were assigned to a large breeding range from the coast of Norway in the west, across to eastern Russia in the east. The highest concentration of origin assignments was located in a wide band stretching from southern Finland to northern Ukraine and across to central Russia (Figure 2).

### 3.3 Is migration distance related to the prevalence and intensity of infection?

**Figure 3:**
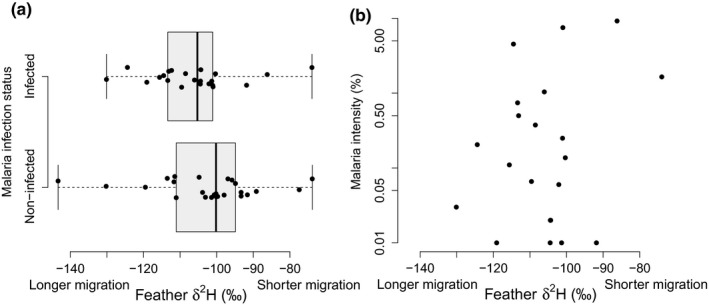
Relationship between migration distance (*δ* ^2^H_f_ ‰) and WW1 (a) prevalence (infected: *n* = 22; noninfected: *n* = 25) and (b) intensity of infection for male willow warblers wintering in Zambia (*n* = 21)

When using generalized linear models, autumn migration distance (*δ* ^2^H_f_) was unrelated to either the prevalence or intensity of malaria infection for willow warbler males at the Zambian nonbreeding site (Figure 3, Table 1). Moreover, probabilistic assignments using *δ* ^2^H_f_ indicated that infected individuals may have originated from across much of the western part of their breeding range, from southern Finland in the west, to southwest and south‐central Russia in the east (Figure 2a). Northwestern Russia near the northern Ural Mountains was also an area where a high number of infected willow warblers potentially originated. The region with the highest concentration of potential origins of noninfected birds was located in a wide band stretching from Estonia south to eastern Poland and east through Belarus, northern Ukraine, southwestern Russia, and northern Kazakhstan (Figure 2b). Figure 2c shows the differences in origins between the scaled rasters of infected and noninfected willow warblers, indicating more northern origins for infected birds. The test for spatial autocorrelation between the spatial processes in the assignments of birds with and without malaria was significant (*F*_1,32.5_ = 29.7, *p* < 0.001, *R* = 0.69), indicating a positive relationship between the origins of infected and noninfected birds. However, despite the visual pattern in Figure 2c, the spatial difference between the origins of infected versus noninfected birds resulted in only slightly longer estimated migration distances for infected birds (~214 km; see Methods2).

**Table 1:** Generalized linear model results testing for relationships between migration distance *δ* ^2^H_f_ (‰), and WW1 prevalence and intensity for male willow warblers wintering in Zambia. Capture date is included as a covariate in both models. Model results include variance inflation factors (VIF)

	Malaria prevalence				Malaria intensity			
	*β*	CI	*p*	VIF	*β*	CI	*p*	VIF
(Intercept)	−3.24	−8.44 to 1.45	0.189		6.11	0.47 to 11.58	0.039	
δ^2^H_f_	−0.01	−0.06 to 0.03	0.542	1.047	0.04	−0.01 to 0.09	0.116	1.006
Capture date	0.04	−0.01 to 0.09	0.135	1.047	−0.04	−0.11 to 0.02	0.276	1.006
Observations	47				21			
*R* ^2^/adj. *R* ^2^	0.069/0.092				0.526/0.547			

### 3.4 Malaria infection and individual condition

We found no relationship between the prevalence or intensity of malaria infection and circulating corticosterone, scaled mass index, or flight feather quality (Table 2).

**Table 2:** Generalized linear model results testing for relationships between corticosterone, scaled mass index, feather quality, and WW1 prevalence and intensity for male willow warblers wintering in Zambia. Capture date is included as a covariate in both models. Model results include variance inflation factors (VIF)

	Malaria prevalence				Malaria intensity			
	*β*	CI	*p*	VIF	*β*	CI	*p*	VIF
(Intercept)	−15.93	−40.51 to 4.57	0.155		21.06	−5.10 to 70.12	0.228	
Corticosterone	−0.39	−1.12 to 0.24	0.249	1.111	0.5	−1.21 to 2.16	0.53	1.303
Scaled mass index	1.56	−0.78 to 4.29	0.215	1.119	−2.38	−7.99 to 0.73	0.243	1.603
Feather quality (PC1)	−0.44	−1.12 to 0.13	0.155	1.053	0.42	−0.86 to 1.65	0.474	1.326
Capture date	0.07	0.01 to 0.14	0.045	1.067	−0.04	−0.16 to 0.05	0.464	1.116
Observations	36				15			
*R* ^2^/adj. *R* ^2^	0.239/0.321				0.624/0.673			

## 4 DISCUSSION

Using generalized linear models, we found no relationship between migration distance (*δ* ^2^H_f_) and either avian malaria prevalence or intensity in male willow warblers sampled on their nonbreeding grounds in Zambia. By contrast, spatial assignments found that infected individuals had more northerly breeding ground origins than noninfected individuals; however, this spatial variation resulted in only a small difference in migration distance (~214 km). These results do not support any of the proposed mechanisms driving host–parasite dynamics in migratory species: migratory culling, immunity/migration trade‐offs, or parasite exposure. In line with previous studies, we also found no association between malaria prevalence or intensity and indices of individual body condition (assessed via corticosterone, scaled mass index and feather quality; Sorensen et al., 2016). This is consistent with the hypothesis that the costs of chronic malaria infection are not always detectable over the short term (Asghar, Hasselquist, & Bensch, 2011; Asghar et al., 2015; Lapointe et al., 2012). However, since song was used to entice birds toward mist nets, our sample may be biased toward individuals in better body condition. Individuals with high infection intensity may have reduced movements, making them difficult to catch, or may be quickly removed from the population via parasite‐induced mortality (Valkiūnas, 2004); this could potentially reduce our power to detect any costs of high infection intensity. These considerations highlight the difficulty of studying parasite infections in wild bird populations, even in areas of active parasite transmission (Sorensen et al., 2016).

Despite the potential for migration distance to influence susceptibility to disease and intensity of disease in long‐distance migratory birds on their African wintering grounds, we found no correlative evidence of such effects. One possible interpretation is that variation in migration distance was not large enough to detect existing relationships in this study population. Breeding origin assignments suggest that migration distance varied by a maximum of ~3,500 km (e.g., birds breeding in arctic Russia vs. southern Ukraine) for the birds in this study. However, most individuals were consistent with origins from a wide band stretching from southern Finland to northern Ukraine and across to central Russia (Figure 2), suggesting a ~1,500 km range in migration distance for the majority of birds in this study. Given that migration distance across populations may vary by as much as 9,000 km for willow warblers (Cramp & Perrins, 1994), subsequent work should seek to incorporate the extremes of individual variation in migration distance. East Africa, where willow warbler subspecies *P*. *t*. *yakutensis* (13,000 km migration distance one way) and *acredula* (8,000 km migration distance one way) likely coexist (Sokolovskis et al., 2018), is a promising location to investigate possible effects of large scale variation in migration distance. However, since future changes to migration distances are likely to occur in small incremental steps, understanding the influence of small variation in migration distance is also important for predictive population dynamics.

Our study was confined to males; however, females may be more susceptible to the possible negative effects of longer migration distances. In most migratory passerine species, females invest more heavily in reproduction through egg production, incubation, and chick feeding duties than males (Ricklefs, 1974). Therefore, longer autumn migration distances may have stronger deleterious effects on females following an energetically taxing breeding season. In addition, juveniles making the journey for the first time may also be more susceptible to the negative effects of longer migrations; however, willow warblers could not be aged upon arrival in Zambia since the typical indicators of age (plumage/cranium) are no longer reliable on the wintering grounds. Our sample was almost entirely restricted to males, likely because we used playback to entice birds toward mist nets and males are the most aggressive sex during the nonbreeding period. Alternatively, it is possible that willow warblers demonstrate sex segregation on their wintering grounds, perhaps generating a male‐based population sex ratio at our study site (Catry, Campos, Almada, & Cresswell, 2004). Whether migration distance is more important for disease dynamics in certain sexes or age classes is an important question for future research, since such a contrast would have clear implications for understanding species population dynamics in the face of deteriorating environmental conditions in both hemispheres (Dirzo et al., 2014; Pecl et al., 2017).

Willow warblers were only infected with the *H. palloris* (lineage WW1) in Zambia, which is transmitted on African wintering grounds rather than breeding grounds (Bensch & Akesson, 2003). The absence of breeding‐transmitted WW2 (*H. majoris*) in the Zambian population may have been because birds with WW2 infections died during migration (Bradley, 2005); because WW2 infections may decline to undetectable levels by the time birds arrive at their wintering grounds; because WW2 was not present on the breeding grounds of this population during the summer preceding this study, which can occur in some years (Bensch & Akesson, 2003); or because WW2 simply does not occur on the breeding range of the Zambian wintering birds (Nilsson et al., 2016). Taken together, then, two lines of evidence suggest that malaria parasite exposure should be similar across individuals in this study irrespective of migration distance (Gutiérrez et al., 2017): (a) the absence of breeding ground transmitted WW2 in this population and (b) the observed variation in migration travel distances occurring over the breeding grounds, rather than wintering grounds, since all birds originated from the same wintering site. Therefore, the trade‐off between migration and immunity, rather than parasite exposure, is the likely mechanism tested in this study. This suggests that individual variation in migration distance, along with its associated energy requirements, is unlikely to influence malaria infection prevalence or intensity, at least on the scale observed in this study. Migratory species are known to have more immunity‐related genes than resident species (Westerdahl et al., 2014) which could help to offset the energy imbalance between immunity and migratory flight; however, a recent study found that immune function does not vary with individual migration distance (Kelly et al., 2017).

The use of δ^2^H_f_ as a proxy for migration distance, and to delineate probable origins of migratory animals from broad molting (e.g., breeding) ranges, presents several challenges. For instance, similarities in the longitudinal gradient of the δ^2^H_p_ isoscape across large parts of Eurasia hinder our ability to determine more precise origins in this direction using δ^2^H_f_ measurements alone. When the appropriate data are available, probabilistic assignments using δ^2^H_f_ can be more precise when used together with other isotopes or nonisotopic prior information. In this study, genetics were useful in eliminating the far western portion of the breeding range from the assignment to origin analyses; however, a large portion of the breeding range remained under consideration. Further advancements in determining morphometric gradients across the breeding grounds for this species, the use of other stable isotopes (assuming such isoscapes are available) or other variables (e.g., movement vector data) will make assignment to origin analyses more exact (Maggini et al., 2016; Rundel et al., 2013; Van Wilgenburg & Hobson, 2011).

Here, we sought to examine potential factors determining individual infection risk in migratory willow warblers. We found no relationship between migration distance and malaria prevalence or intensity of infection on the nonbreeding grounds using generalized linear models, and only a small difference in migration distance for infected and noninfected birds using spatial assignments. This evidence cautiously suggests that other factors may be more important for determining individual variation in infection risk for migratory birds. Given that recent work has demonstrated that chronic malaria infections have long‐term negative effects on individual lifespan and fitness (Asghar et al., 2015), understanding the factors influencing infection risk and intensity of disease remains an important avenue for future research.

## CONFLICT OF INTEREST

The authors declare no competing interests.

## AUTHOR CONTRIBUTIONS

MCS and CNS conceived the study. TD, MCS, JN, and SJ‐E processed samples in the laboratory. MCS and KJK analyzed the data. MCS drafted the manuscript. All authors contributed to interpretation and writing. All authors approved the final version.

## Data Availability

Data are available on Dryad: https://doi.org/10.5061/dryad.57c0420.
